# Patterns of Ownership and Usage of Wearable Devices in the United States, 2020-2022: Survey Study

**DOI:** 10.2196/56504

**Published:** 2024-07-26

**Authors:** Ashwini Nagappan, Adriana Krasniansky, Madelyn Knowles

**Affiliations:** 1 Department of Health Policy and Management University of California, Los Angeles Los Angeles, CA United States; 2 Rock Health San Francisco, CA United States

**Keywords:** digital health, health equity, adoption, usage patterns, wearable devices, United States, adoption, technology, sociodemographic, survey, health insurance, public health

## Abstract

**Background:**

Although wearable technology has become increasingly common, comprehensive studies examining its ownership across different sociodemographic groups are limited.

**Objective:**

The aims of this study were to (1) measure wearable device ownership by sociodemographic characteristics in a cohort of US consumers and (2) investigate how these devices are acquired and used for health-related purposes.

**Methods:**

Data from the Rock Health Digital Health Consumer Adoption Survey collected from 2020 to 2022 with 23,974 US participants were analyzed. The sample was US Census–matched for demographics, including age, race/ethnicity, gender, and income. The relationship between sociodemographic factors and wearable ownership was explored using descriptive analysis and multivariate logistic regression.

**Results:**

Of the 23,974 respondents, 10,679 (44.5%) owned wearables. Ownership was higher among younger individuals, those with higher incomes and education levels, and respondents living in urban areas. Compared to those aged 18-24 years, respondents 65 years and older had significantly lower odds of wearable ownership (odds ratio [OR] 0.18, 95% CI 0.16-0.21). Higher annual income (≥US $200,000; OR 2.27, 95% CI 2.01-2.57) and advanced degrees (OR 2.23, 95% CI 2.01-2.48) were strong predictors of ownership. Living in rural areas reduced ownership odds (OR 0.65, 95% CI 0.60-0.72). There was a notable difference in ownership based on gender and health insurance status. Women had slightly higher ownership odds than men (OR 1.10, 95% CI 1.04-1.17). Private insurance increased ownership odds (OR 1.28, 95% CI 1.17-1.40), whereas being uninsured (OR 0.41, 95% CI 0.36-0.47) or on Medicaid (OR 0.75, 95% CI 0.68-0.82) decreased the odds of ownership. Interestingly, minority groups such as non-Hispanic Black (OR 1.14, 95% CI 1.03-1.25) and Hispanic/Latine (OR 1.20, 95% CI 1.10-1.31) respondents showed slightly higher ownership odds than other racial/ethnic groups.

**Conclusions:**

Our findings suggest that despite overall growth in wearable ownership, sociodemographic divides persist. The data indicate a need for equitable access strategies as wearables become integral to clinical and public health domains.

## Introduction

Consumers are increasingly integrating wearable technology, which refers to devices that can be worn on the body to capture data [1], into their daily lives [2]. Wearable devices now range from smartwatches to fertility trackers and sleep trackers, serving a variety of health monitoring applications [3,4]. While prior studies have focused on the adoption of wearables within specific populations such as adolescents, older adults, and underserved populations [5-7], large-scale studies examining differences in wearable adoption across sociodemographic characteristics remain limited.

This study aimed to measure wearable ownership by sociodemographic characteristics in a cohort of US consumers. Specifically, a respondent was considered a wearable owner if, at the time of the survey, they reported currently owning a wearable device, even if they did not currently use the device. Additionally, we examined how respondents received their wearable devices and explored relevant health-related uses. The findings provide important insights to guide wearable product development and identify priorities to improve ownership among underrepresented groups.

## Methods

### Study Design

This study aggregated data from 3 consecutive years (2020-2022) of the Rock Health Digital Health Consumer Adoption Survey [8]. The total sample consisted of 23,974 US-based respondents (7,980 in 2020, 7,980 in 2021, and 8,014 in 2022). Survey respondents were not excluded from participating in the study in subsequent years and repeat respondents comprised 3.3% (n=784) of the total cohort. The survey was administered by Toluna, a survey management organization. Toluna used its existing panel, initially recruited via online advertising, to identify participants. Eligible participants were 18 years or older, and the sample was Census-matched each year according to age, race/ethnicity, geographic region, gender, and annual household income.

### Ethical Considerations

In accordance with the Common Rule (Federal Policy for the Protection of Human Subjects, 82 Federal Regulation 7259, January 19, 2017 [9]), this study was exempt from regulations for research with human subjects as the data were deidentified.

### Data Collection and Survey Questions

Surveys were digitally administered through Toluna’s platform, and respondents used their personal desktop, laptop, smartphone, or tablet to complete the survey in English. The survey encompassed 4 main domains: (1) sociodemographic factors, (2) health status, (3) adoption of digital health tools (eg, telemedicine, wearables), and (4) attitudes toward and perceptions of digital health technology. For this study, we focused on questions about wearable technology adoption.

### Measures

The primary outcome was wearable ownership as measured by the survey question, “Do you own a wearable device or smartwatch (note: this excludes smartphones) that helps you track your health? This could include: number of steps/exercise, sleep, heart rate, or blood pressure.” Covariates included sociodemographic characteristics (eg, age, gender, race/ethnicity, income level, and educational level) and self-reported health status. The secondary analysis was exploratory, delving into the cohort of wearable owners to investigate the source of their devices and their reasons for using wearables. Respondents were asked: “Do or did you use your wearable device or smartwatch for any of the purposes listed below? Select all that apply.”

### Data Analysis

We performed a descriptive analysis of the pooled sample and examined associations with wearable ownership. The *χ*^2^ test was used to assess variation across covariates and multivariate logistic regression was used to identify factors associated with wearable ownership. Additional analyses explored how respondents acquired their wearables and what they used the devices for. Analyses were conducted in Stata v.16.

## Results

The analytic sample included 23,974 survey respondents, including 13,295 wearable nonowners and 10,679 wearable owners (Table 1). Younger respondents (18-44 years) exhibited higher ownership rates, while nonowners skewed toward the older demographic (55+ years). An income gradient emerged, with those earning less than US $25,000 annually representing a smaller fraction of owners (10.5%) than nonowners (25.8%), with ownership rates increasing with income. Advanced degree holders represented 31.4% of wearable device owners versus 13.5% of nonowners.

Ownership varied by rurality, with 45.6% of owners in urban areas compared to 12.6% in rural areas. Nonowners were more evenly distributed, with 27.8% in urban areas and 23.4% in rural areas. Men represented a slightly higher proportion of owners than women. Health insurance coverage emerged as another differentiator; 41.2% of wearable owners had employment-based insurance and 21.5% had private insurance, while nonowners were more likely to be covered by Medicare, Medicaid, or to be uninsured. Ownership also varied across racial and ethnic groups, with higher proportions of non-Hispanic Black, non-Hispanic Asian, and Hispanic/Latine respondents in the owner group. Nearly one-third of owners self-reported excellent health, which was over twice the rate among nonowners.

Multivariable logistic regression was used to model the relationship between sociodemographics and wearable ownership, controlling for covariates (Table 2). Wearable device ownership increased from 2020 to 2022. Compared to the age group of 18-24 years, all other age groups showed decreased odds of ownership, especially those 65 years and older. Higher income and education significantly increased ownership odds, with those earning US $200,000 and above per year and those with advanced degrees showing more than double the odds compared to those of the comparator groups.

**Table 1:** Respondent characteristics, by wearable ownership, 2020-2022 (N=23,974).

Characteristic			Nonowners (n=13,295), n (%)		Owners (n=10,679), n (%)		*P* value^a^	
**Age (years)**								<.001
	18-24	1340 (10.1)		1424 (13.3)				
	25-34	1743 (13.1)		2574 (24.1)				
	35-44	1457 (11.0)		2586 (24.2)				
	45-54	2112 (15.9)		1778 (16.6)				
	55-64	2677 (20.1)		1196 (11.2)				
	65+	3966 (29.8)		1121 (10.5)				
**Annual household income (US $)**								<.001
	<25,000	3428 (25.8)		1117 (10.5)				
	25,000-34,999	1401 (10.5)		758 (7.1)				
	35,000-49,999	1854 (13.9)		1019 (9.5)				
	50,000-74,999	2365 (17.8)		1627 (15.2)				
	75,000-99,999	1381 (10.4)		1493 (14.0)				
	100,000-149,999	1501 (11.3)		2072 (19.4)				
	150,000-199,999	549 (4.1)		1128 (10.6)				
	≥200,000	717 (5.4)		1443 (13.5)				
	Prefer not to say	99 (0.7)		22 (0.2)				
**Education**								<.001
	Less than high school	430 (3.2)		151 (1.4)				
	High school graduate (includes equivalency)	3376 (25.4)		1560 (14.6)				
	Some college, no degree	3232 (24.3)		1680 (15.7)				
	Associate degree	1519 (11.4)		1227 (11.5)				
	Bachelor degree	2897 (21.8)		2677 (25.1)				
	Master degree	1244 (9.4)		2297 (21.5)				
	PhD	160 (1.2)		306 (2.9)				
	Graduate or professional degree (eg, MD, JD)	386 (2.9)		749 (7.0)				
	Prefer not to say	51 (0.4)		32 (0.3)				
**Rurality**								<.001
	Rural	3105 (23.4)		1346 (12.6)				
	Suburban	6496 (48.9)		4461 (41.8)				
	Urban	3694 (27.8)		4872 (45.6)				
**Gender**								<.001
	Woman	6840 (51.4)		5116 (47.9)				
	Man	6330 (47.6)		5476 (51.3)				
	Other	77 (0.6)		74 (0.7)				
	Prefer not to disclose	48 (0.4)		13 (0.1)				
**Health insurance**								<.001
	Employment-based	3559 (26.8)		4405 (41.2)				
	Private purchase	1375 (10.3)		2294 (21.5)				
	Medicare (over the age of 65)	3995 (30.0)		1559 (14.6)				
	Medicaid	2105 (15.8)		1287 (12.1)				
	Other public	454 (3.4)		375 (3.5)				
	Other	253 (1.9)		150 (1.4)				
	Uninsured	1052 (7.9)		374 (3.5)				
	I don’t know	502 (3.8)		235 (2.2)				
**Race/ethnicity**								<.001
	NH^b^-White	8941 (67.3)		6443 (60.3)				
	NH-Black or African-American	1373 (10.3)		1310 (12.3)				
	NH-American Indian or Alaska Native	53 (0.4)		58 (0.5)				
	NH-Asian	603 (4.5)		570 (5.3)				
	NH-Hawaiian Native or Pacific Islander	38 (0.3)		59 (0.6)				
	NH-Other	69 (0.5)		24 (0.2)				
	Multiracial, NH	257 (1.9)		230 (2.2)				
	Hispanic/Latine	1881 (14.1)		1962 (18.4)				
	Prefer not to say	80 (0.6)		23 (0.2)				
**Self-reported health status**								<.001
	Very poor	160 (1.2)		36 (0.3)				
	Poor	958 (7.2)		238 (2.2)				
	Moderate	3384 (25.5)		1609 (15.1)				
	Good	6913 (52.0)		5386 (50.4)				
	Excellent	1880 (14.1)		3410 (31.9)				

^a^*P* values were calculated using *χ*^2^ tests to compare the distributions of each variable across the two groups. Due to the large sample sizes, statistically significant *P* values may be observed even for minor differences; therefore, *P* values should be interpreted with caution and in the context of practice relevance beyond statistical significance.

^b^NH: non-Hispanic.

**Table 2:** Multivariable analysis of wearable ownership and sociodemographic characteristics, 2020-2022 (N=23,974).

Variable		Adjusted odds ratio (95% CI)	*P* value
**Year (reference: 2020)**			
	2021	1.16 (1.08-1.25)	<.001
	2022	1.27 (1.18-1.36)	<.001
**Age (years) (reference: 18-24)**			
	25-34	0.98 (0.88-1.09)	.68
	35-44	0.90 (0.80-1.01)	.06
	45-54	0.52 (0.46-0.58)	<.001
	55-64	0.32 (0.28-0.36)	<.001
	65+	0.18 (0.16-0.21)	<.001
**Annual income (US $) (reference: <50,000)**			
	50,000-99,999	1.62 (1.50-1.75)	<.001
	100,000-149,999	2.02 (1.83-2.23)	<.001
	150,000-199,999	2.73 (2.40-3.12)	<.001
	≥200,000	2.27 (2.01-2.57)	<.001
**Education (reference: ≤high school diploma)**			
	Some college	1.27 (1.17-1.38)	<.001
	Bachelor’s degree	1.44 (1.32-1.59)	<.001
	Advanced degree	2.23 (2.01-2.48)	<.001
**Rurality (reference: urban)**			
	Rural	0.65 (0.60-0.72)	<.001
	Suburban	0.72 (0.68-0.77)	<.001
Gender: women (reference: men)		1.10 (1.04-1.17)	.001
**Health insurance (reference: employment-based)**			
	Private purchase	1.28 (1.17-1.40)	<.001
	Medicare (over the age of 65)	1.06 (0.95-1.19)	.29
	Medicaid	0.75 (0.68-0.82)	<.001
	Uninsured	0.41 (0.36-0.47)	<.001
**Race/ethnicity (reference: NH^a^-White)**			
	NH-Black or African-American	1.14 (1.03-1.25)	.008
	NH-American Indian or Alaska Native	1.04 (0.68-1.57)	.87
	NH-Asian	0.82 (0.72-0.94)	.004
	NH-Hawaiian Native or Pacific Islander	1.59 (1.03-2.45)	.04
	NH-Other	0.62 (0.38-1.03)	.07
	Multiracial, NH	1.02 (0.84-1.25)	.82
	Hispanic/Latine	1.20 (1.10-1.31)	<.001
**Health status (reference: excellent)**			
	Very poor	0.30 (0.21-0.45)	<.001
	Poor	0.36 (0.31-0.43)	<.001
	Moderate	0.53 (0.48-0.58)	<.001
	Good	0.66 (0.62-0.72)	<.001

^a^NH: non-Hispanic.

Living in rural areas was associated with 35% lower ownership odds, and women demonstrated slightly higher odds than men. Compared to employment-based plans, private insurance increased ownership odds. Conversely, being uninsured significantly reduced the odds of wearable ownership, as did being covered by Medicaid. Relative to non-Hispanic White respondents, non-Hispanic Black and Hispanic/Latine respondents had higher ownership odds, whereas non-Hispanic Asian respondents showed lower odds. Excellent self-reported health predicted the highest odds of ownership.

The majority of owners purchased their devices (61.1%), followed by 24.1% who received them as gifts (Table 3). Smaller proportions obtained devices from health care providers, employers, or insurance companies. Wearable owners primarily used their devices for fitness and wellness; top-use cases included physical activity (56.6%), fitness training (55.4%), and losing weight (43.8%). Approximately one-third of respondents reported using their wearable to manage a diagnosed condition (Table 3).

**Table 3:** Source of wearable device and top health-related uses of wearable devices among wearable owners (N=10,679).

Source and use		Respondents, n (%)
**Source of wearable device**		
	I purchased it myself	6521 (61.1)
	It was a gift	2574 (24.1)
	It was offered to me by my doctor/clinician	862 (8.1)
	It was offered to me by my insurance company	376 (3.5)
	It was offered to me by my employer	123 (1.2)
	None of these	223 (2.1)
**Top health-related uses of wearable devices**		
	More physical activity	6049 (56.6)
	Fitness training	5911 (55.4)
	Lose weight	4682 (43.8)
	Sleep better	3834 (35.9)
	Manage diagnosed condition	3643 (34.1)
	Manage stress	3075 (28.8)

## Discussion

### Principal Findings

Despite steadily increasing wearable ownership from 2020 to 2022 among US consumers, sociodemographic disparities persist related to age, income, education, and residence. Ownership skews toward younger, more affluent, and highly educated respondents living in urban areas. This is consistent with prepandemic findings [2,10], suggesting continuity in the digital divide [11]. This unequal access impacts the potential benefits of wearables on health promotion and health outcomes [12-15].

Surprisingly, non-Hispanic Black and Hispanic/Latine respondents have slightly higher ownership odds than non-Hispanic White respondents. If corroborated by future research indicating active usage among these communities, this finding suggests that wearables can be harnessed to monitor chronic conditions prevalent in these communities, such as hypertension and diabetes [16,17]. The inclination of wearable ownership among these groups presents an opportunity to inform the design and development of new wearable technologies [18].

Reliance on direct-to-consumer channels (eg, direct purchase or gifts) for acquiring wearables underscores the discretionary nature of wearable purchase and signals a possible relationship between health consciousness and wearable ownership [19]. The self-motivated nature of acquisition points to the importance of consumer preferences in uptake and indicates a potential barrier to access among those with limited discretionary means. This signals a need to improve equitable access through alternative distribution pathways.

As wearables are considered for clinical care [20] and public health surveillance applications [21-23] such as communicable respiratory (eg, COVID-19) and infectious (eg, dengue) diseases, it will be critical to continue to track ownership patterns and address biases in the wearable user population related to age, income, education, and area of residence [24,25]. Thus, effective wearable-based public health surveillance efforts must actively mitigate such biases within the current wearable-owning population and increase wearable uptake among subgroups reporting lower rates of ownership. In the near term, acknowledging and adjusting for biases is essential. This requires recognizing barriers that lead to uneven ownership. Over the longer term, there is an opportunity to address barriers to wearable ownership, which can allow the user cohort to gradually better represent the general population.

### Limitations

Study limitations include sampling bias, as the survey undersamples those without digital devices (eg, smartphones, tablets, or computers), regular internet connectivity, and non-English speakers. While the study used a pooled sample across 3 years, a small proportion of respondents were surveyed in multiple waves, which may affect comprehensiveness. Further, the study focuses on ownership, not wearable adoption, impacting findings related to active usage patterns of wearables. Additionally, respondents who indicated ownership may or may not own more than one device, which could impact the responses to subsequent questions.

### Conclusion

This study provides an updated benchmark on wearable device ownership among US consumers, highlighting sociodemographic disparities in ownership across age, income, education, and residence. While some traditionally disadvantaged groups demonstrate higher ownership rates, other findings reflect persisting disparities, painting a mixed picture of progress toward a more equitable wearable distribution. This underscores the need for further research, particularly into higher ownership rates among minority groups. Future research may also explore reasons for nonownership among wearable nonowners, such as lack of interest, privacy concerns, cost, or other barriers. Understanding these differences is critical for designing inclusive wearable products and business models, and for ensuring the appropriate use of wearable data in health care and public health research by accounting for biases in sample representation.

## Abbreviations

OR: odds ratio
